# New onset refractory status epilepticus diagnosed in the second trimester: A case report

**DOI:** 10.1177/1753495X251386551

**Published:** 2025-10-15

**Authors:** Vesna Sokol Karadjole, Dareen AlShaer, John W Snelgrove, Laurence Sophie Carmant, Ginette Moores

**Affiliations:** 1 Maternal-Fetal Medicine, Obstetrics and Gynaecology, Mount Sinai Hospital, 574414 University of Toronto, Toronto, Canada; 2 Division of Neurology, Department of Medicine, 7938 University of Toronto, Toronto, Ontario, Canada; 3 Institute of Health Policy, Management and Evaluation, 7938 University of Toronto, Toronto, Canada

**Keywords:** NORSE, pregnancy, antiseizure medications, neonatal outcome, case report

## Abstract

New-onset refractory status epilepticus (NORSE) is a rare condition in which a previously healthy individual develops refractory seizures without an identifiable cause. In pregnancy, management is particularly challenging due to the need to control seizures while minimizing teratogenic risk for the fetus. We report a 22-year-old woman who developed NORSE at 19 weeks’ gestation following recurrent tonic-clonic seizures. Treatment included multiple antiseizure medications: levetiracetam, oxcarbazepine, lacosamide, clobazam, and lamotrigine. Due to super-refractory status, she required intubation and sedation with propofol and midazolam and was extubated once seizure-free. Following a breakthrough seizure and suicide attempt, levetiracetam was replaced with brivaracetam. Fetal growth and biophysical profile remained appropriate on serial surveillance. She underwent term induction of labor, delivering a healthy neonate without signs of withdrawal. This is the first reported case of second-trimester NORSE with favorable perinatal outcomes, underscoring the need for a multidisciplinary approach to balance seizure control and fetal safety.

## Introduction

New-onset refractory status epilepticus (NORSE) is rare and characterized by status epilepticus unresponsive to standard treatment without an apparent structural, toxic, or metabolic cause.^1^ If an autoimmune or infectious trigger is later identified, it is still classified as NORSE; if no cause is found, it is termed cryptogenic.^2^

NORSE accounts for 20% of refractory status epilepticus (RSE) cases and carries a high risk of neurological morbidity and mortality.^3^ Though uncommon, it can affect young individuals, including pregnant women, where altered antiseizure medication (ASM) metabolism and teratogenicity complicate management.

Only one case of NORSE in early pregnancy has been reported, requiring vagal nerve stimulation for seizure control.^4^

## Case presentation

A healthy 22-year-old woman in her first pregnancy presented to the emergency department at 18 weeks and 6 days of gestation, with a couple of episodes of tonic-clonic seizure lasting less than 2 min. She had no history of epilepsy or seizure risk factors. She was started on levetiracetam 500 mg twice daily (BID). Initial labs, brain imaging, and CSF testing were unremarkable (Tables 1 and 2).

**Table 1.** Maternal serum and cerebrospinal fluid investigations.

Investigations	Findings
*Serum*	* *
Metabolic panel (including Zn, Cu, Se, Mg, Ca, and phosphate)	Normal
Autoimmune encephalitis antibodies (anti-AMPA-1, AMPA-2, Anti- GABA, Anti-DPPX, Anti-VGKC antibodies)	Negative
Neuronal antibodies (including Hu, Ri, and Yo antibodies, GAD 65)	Negative
NMDA receptor antibodies	Negative
TPO antibodies	Negative
*Cerebral spinal fluid (Day 2 of admission)*	
Total cells counted	13 (100% lymphocytes)
Protein	0.39 mmol/L (0.15- 0.45)
Glucose	3.2 mmol/L/L (2.2-3.9)
CSF virology (including CMV, VZV, HSV 1 and 2, enterovirus and adenovirus)	Not detected
CSF infectious (including mycobacteria, cryptococcus, fungal, gram stain, and culture)	Negative
*Cerebral spinal fluid (Day 6 of admission)*	
Total cells counted	16 (97% lymphocytes)
Protein	0.22 g/L (0.15-0.45)
Lactate	2.1 mmol/L (<2.8)
Glucose	3.6 mmol/L (2.2-4.4)
CSF virology (including CMV, enterovirus, VZV, HSV 1 and 2, HHV 6, HPeV, WNV, and other arbovirus)	Negative
CSF infectious (including *Escherichia coli*, *Haemophilus influenzae, Listeria monocytogenes, Neisseria meningitidis, Streptococcus agalactiae, Streptococcus pneumoniae, Cryptococcus, Borrelia burgdorferi*)	Negative
Autoimmune encephalitis antibodies (Anti-AMPA-1, AMPA-2, Anti- GABA, Anti-DPPX, Anti-VGKC antibodies)	Negative
Paraneoplastic antibodies (Hu, Ri, Yo, Ma1, amphiphysin, CV2, recoverin, SOX1, titin, GAD 65, Zio4, Tr (DNER)	Negative
NMDA receptor antibodies	Negative

*Note:* Zn: zinc; Cu: copper; Se: selenium; Mg: magnesium; Ca: calcium; anti-AMPA: anti-glutamate receptor 1 (AMPA subtype); anti-GABA: anti-gamma-aminobutyric acid; anti-DPPX: anti-dipeptidyl-peptidase-like protein 6; anti-VGKC: anti-voltage-gated potassium channel; GAD 65: glutamic acid decarboxylase 65; NMDA: N-methyl-D-aspartate; TPO: thyroid peroxidase; CSF: cerebrospinal fluid; CMV: cytomegalovirus; VZV: varicella zoster virus; HSV: herpes simplex virus; HHV 6: human herpesvirus 6; HPeV: human parechovirus; WNV: West Nile virus; SOX1: anti-Sry-like high mobility group box; Tr (DNER) : Tr/anti-Delta/Notch-like epidermal growth factor-related receptor antibody.

**Table 2.** Imaging and EEG findings.

Examination	Findings
MRI/MRV head	No venous sinus thrombosis or other abnormalities.
Initial EEG (Day 2 of admission)	Two ∼1-min electrographic seizures from the left temporal region with secondary generalization; no clinical signs observed. Frequent interictal discharges over left temporal/frontotemporal areas. Background slow with polymorphic delta activity.
Pelvic ultrasound	No evidence of an ovarian teratoma.
CT chest	No evidence of an intrathoracic teratoma.
MRI abdomen	No evidence of malignancy or teratoma.
Obstetric ultrasound (transabdominal)	Fetal anatomy normal; fetal growth appropriate.
Final EEG prior to discharge (Day 34 of admission)	Normal EEG in wakefulness and sleep.

*Note:* MRI/MRV: magnetic resonance imaging/magnetic resonance venography; EEG: electroencephalogram; CT: computed tomography.

Despite treatment, she continued to have focal seizures with automatisms. Levetiracetam was increased and oxcarbazepine and lacosamide were added sequentially. Electroencephalogram (EEG) showed left temporal seizures without clinical correlation. Given the subclinical seizures refractory to several ASM, she was transferred to a tertiary care center for continuous EEG monitoring.

By Day 9, she experienced worsening tonic-clonic seizures requiring intubation and sedation with propofol and midazolam. Her ASM regimen included levetiracetam 2 g twice daily, clobazam 20 mg twice daily, lacosamide 2000 mg twice daily, oxcarbazepine 600 mg twice daily, and lamotrigine 50 mg twice daily. Repeat infectious work-up and malignancy screening were negative (Table 1).

On Day 12, she was started on intravenous immunoglobulin and a five-day course of intravenous methylprednisolone, followed by oral prednisone (1 mg/kg). EEG showed multifocal discharges without seizures. Sedation was weaned, but stopping midazolam (Day 23) led to recurrent electrographic seizures. Midazolam was resumed and oxcarbazepine increased to 900 mg twice daily. By Day 26, she was extubated and seizure-free.

On Day 29, the woman was transferred to the tertiary obstetric center, fetal ultrasound showed normal growth and biophysical parameters (Table 2). One week later, she was discharged home with ASM therapy and slow prednisone taper.

### Outpatient course and delivery

She remained seizure-free initially but developed ASM-related side effects (ataxia, drowsiness). Lamotrigine was gradually discontinued given its subtherapeutic dose and concern for multiple sodium channel blockade.

One month later, she had a breakthrough seizure following fasting for her oral glucose tolerance test. Shortly after, she had mood dysregulation with a suicide attempt by overdosing on her ASM. Psychiatry attributed mood instability to levetiracetam and corticosteroids. Levetiracetam was switched to brivaracetam (150 mg twice daily) in addition to ongoing prednisone taper with clinical improvement.

A multidisciplinary meeting was held for delivery planning. At 38 weeks, she underwent an uncomplicated induced vaginal delivery of a healthy male neonate. Due to prolonged steroid use in pregnancy, stress dose steroids were administered. Postpartum course was uneventful. At six-week follow-up with neurology, maternal fetal medicine, and perinatal mental health, she reported two additional seizures, triggered by sleep deprivation and missed ASM doses.

## Discussion

This is the first reported case of second-trimester NORSE with a good perinatal outcome.

RSE is a medical emergency whereby recurrent seizures fail to respond to initial treatment with ASM. While rare during pregnancy, it is of concern as it is associated with increased risks of fetal and maternal mortality.^5,6^

Two studies have linked tonic-clonic seizures during pregnancy to increased risk of preterm birth and small for gestational age.^7,8^ In one case, seizures during labor were demonstrated to prolong uterine contraction, compromising placental blood flow and causing fetal hypoxia.^9^ Other risks of maternal seizures include fetal injury, placental abruption, or intrauterine fetal death due to maternal trauma sustained during a seizure.^5^ Unfortunately, limited research has been conducted to assess the direct impact of maternal seizures on fetal well-being, and there is a lack of information regarding the frequency or duration of seizures that may endanger the fetus.

In nonpregnant women, early immunotherapy is recommended for NORSE, ideally within 72 h of SE onset.^10^ No pregnancy-specific guidelines exist; consensus suggests applying standard SE management principles during pregnancy. The International League Against Epilepsy advises prompt and aggressive treatment of tonic-clonic SE in pregnancy.^11^

In our case, the initial delay in immunotherapy likely resulted from diagnostic uncertainty and initial concerns regarding fetal safety prior to the involvement of maternal–fetal medicine. EEG improved rapidly thereafter, enabling sedation wean and extubation.

Five ASMs were ultimately required, with close multidisciplinary monitoring supporting fetal well-being.

Seizure management in pregnancy must balance fetal drug risks against the dangers of uncontrolled seizures for both mother and fetus. Pregnancy-induced pharmacokinetic changes—especially in lamotrigine, levetiracetam, and oxcarbazepine—may necessitate frequent dose adjustments.^12^ Lamotrigine, despite favorable safety, requires slow titration over several weeks, limiting its role in SE. Additionally, safe intravenous ASM options in pregnancy are limited.

Benzodiazepines are the first-line treatment for SE with levetiracetam, valproic acid and phenytoin representing the second-line agents. Levetiracetam is preferred over valproic acid and phenytoin due to a lower teratogenic profile.^13^ Polytherapy increases teratogenicity risk, particularly if valproic acid is included.^14,15^ For RSE requiring sedation, propofol and midazolam are considered safe in pregnancy.^16^

Our patient's suicide attempt illustrates psychiatric vulnerability among pregnant individuals with epilepsy. One prospective study found increased rates of depression, anxiety, and suicidal ideation in this population.^17^ Maternal anxiety is also linked to adverse neurodevelopmental outcomes.^18^ Levetiracetam, in particular, has been associated with behavioral side effects, necessitating psychiatric support and mood screening in prenatal epilepsy care. Limited safety data exist for brivaracetam in pregnancy, but current evidence shows no specific teratogenic or fetal risks.^19^ After discussing risks and benefits, our patient was successfully switched from levetiracetam to brivaracetam. This underscores the need for continued research on newer ASMs during pregnancy.^20^

NORSE in pregnancy requires prompt recognition, early immunotherapy, and multidisciplinary coordination. Management should follow established SE protocols with individualized ASM risk–benefit assessment. With close monitoring, favorable outcomes are achievable.
